# Music and Language in Social Interaction: Synchrony, Antiphony, and Functional Origins

**DOI:** 10.3389/fpsyg.2019.01514

**Published:** 2019-07-02

**Authors:** Nathan Oesch

**Affiliations:** ^1^Music and Neuroscience Lab, Department of Psychology, The Brain and Mind Institute, Western University, London, ON, Canada; ^2^Cognitive Neuroscience of Communication and Hearing (CoNCH) Lab, Department of Psychology, The Brain and Mind Institute, Western University, London, ON, Canada

**Keywords:** synchrony, animal communication, music evolution, evolutionary linguistics, social complexity hypothesis, social bonding, language evolution, evolutionary musicology

## Abstract

Music and language are universal human abilities with many apparent similarities relating to their acoustics, structure, and frequent use in social situations. We might therefore expect them to be understood and processed similarly, and indeed an emerging body of research suggests that this is the case. But the focus has historically been on the individual, looking at the passive listener or the isolated speaker or performer, even though social interaction is the primary site of use for both domains. Nonetheless, an important goal of emerging research is to compare music and language in terms of acoustics and structure, social interaction, and functional origins to develop parallel accounts across the two domains. Indeed, a central aim of both of evolutionary musicology and language evolution research is to understand the adaptive significance or functional origin of human music and language. An influential proposal to emerge in recent years has been referred to as the social bonding hypothesis. Here, within a comparative approach to animal communication systems, I review empirical studies in support of the social bonding hypothesis in humans, non-human primates, songbirds, and various other mammals. In support of this hypothesis, I review six research fields: (i) the functional origins of music; (ii) the functional origins of language; (iii) mechanisms of social synchrony for human social bonding; (iv) language and social bonding in humans; (v) music and social bonding in humans; and (vi) pitch, tone and emotional expression in human speech and music. I conclude that the comparative study of complex vocalizations and behaviors in various extant species can provide important insights into the adaptive function(s) of these traits in these species, as well as offer evidence-based speculations for the existence of “musilanguage” in our primate ancestors, and thus inform our understanding of the biology and evolution of human music and language.

## Introduction

Music and language are universal human capacities with many commonalities relating to their acoustics, structure, and frequent implementation in social situations (Brown, 1991; Pierrehumbert, 1991; Brown and Jordania, 2013; Trehub et al., 2015). Therefore, it might be predicted that they would both be understood and processed in a similar fashion, and an emerging body of research suggests that this is the case (Patel, 2008; Jackendoff, 2009; Koelsch, 2012). However, the focus has historically been on the individual, analyzing the inactive recipient, solitary musician or talker. To date, research has not generally studied how listening and producing are integrated within successful interpersonal coordination, even though interaction is certainly the primary site of use for both domains. Nor has research attempted to explain those aspects of both domains, which may have a common evolutionary history (although see Ravignani et al., 2014, 2017; Filippi, 2016; Hadley and Pickering, 2018 for a few recent exceptions). An important goal of emerging research is to compare music and language in terms of acoustics and structure, social interaction, and functional origins to develop parallel accounts across the two domains.

In terms of acoustics and structure, music and language have many similarities. In music, rhythm establishes an isochronous beat, but also maintains a definite syllable rate in speech, such as in speech tempo (Laver, 2012). Pitch carries the advancement of melody in music, but also underlies prosody in expression and conversation in language. Both music and language consist of many repeated units, with an infinite number of combinations, which are often hierarchically organized, and frequently performed in coordination with other people (Lerdahl and Jackendoff, 1983). Both are therefore rule-based, can be conveyed in written form, found in all known cultures (Huron, 2001; Fritz et al., 2009; Crystal, 2010; Brown and Jordania, 2013; Savage et al., 2015), and often combined (e.g., in singing). Such parallels have led some to argue that the two systems share biological foundations (Brown, 2000; Fitch, 2006).

In terms of interaction, music, and language also have many similarities. People can speak concurrently in prayers or chants, as well as play concurrently in choral, and orchestral works with very accurate timing (Shaffer, 1984; Cummins, 2003). In some cases, language can be used consecutively in dialogue, in a way that is paralleled in musical call-and-response (Stivers et al., 2009). Here, both interlocutors and duettists do not extensively overlap their contributions or leave long gaps between them, but instead take fairly orderly turns (e.g., 40 ms differences between pairs of speakers or duetting musicians; Shaffer, 1984; Cummins, 2003). Moreover, social interaction seems central for regulating this timing. For instance, timing between words is more regular when individuals are speaking in synchrony, compared to when speaking alone (Bowling et al., 2013). In addition, timing appears to depend similarly, on underlying beat perception for both modalities (e.g., Sacks et al., 1974; Ragert et al., 2013).

Nonetheless, there are at least two important distinctions between music and language. First, there is consensus that strict isochronous or rhythmic speech – having units or “beats” that occur at the same time or periodicity – seems artificial and unsustainable in spoken language, where speech tempo is often highly variable (Bowling et al., 2013; Kotz et al., 2018). Second, at least in the normative case, music is generally produced in *synchrony*, where individuals produce events at the same time, while language is produced in *antiphony*, where individuals alternate turns (Ravignani et al., 2014). Thus, both the isochrony and synchrony natural of music, in comparison to their dearth in spoken language, embody the most straightforward distinction between music, and language. That said, there is no perfect division, since tempo in speech can be highly flexible in adult conversation, more periodic in child-directed speech, poetry and oration, and nearly isochronous in rap, chanting, or song (Hawkins, 2014; Menninghaus et al., 2015; Obermeier et al., 2016). Thus, there is a continuum from strict isochrony and synchrony in both music and language, with different styles occupying different places along this spectrum (Kotz et al., 2018).

In terms of origin and function, music and language also have many similarities. Issues that relate to *function* make up one of Tinbergen’s four questions with respect to biological systems: i.e., the adaptive role of a behavior in the environment. The remaining three questions relate to *mechanism*, i.e., the anatomical and physiological causes of a behavior, *phylogeny*, i.e., the behavior’s evolutionary history, and *ontogeny*, i.e., the developmental course of a behavior over the lifespan of an organism (Tinbergen, 1963). Ontogenetic and mechanistic questions explore proximate causes (i.e., “how” questions), while functional and phylogenetic questions address ultimate causes (i.e., “why” questions). Although we still do not know for certain how, when, or why music and language originated in the human species, an influential proposal to emerge in recent years is known as the social brain hypothesis (Dunbar, 1998; Oesch, 2018), and by extension, the social complexity hypothesis (or, more colloquially, the social bonding hypothesis) (Freeberg et al., 2012; Kennedy and Adolphs, 2012; Oesch, 2018). In effect, this proposal states that both human music and language may have evolved for the function of group cohesion in increasingly larger social groups, perhaps underpinned by the neural reward system (Freeman, 2000; Tarr et al., 2014; Oesch, 2018). In particular, the coordinated verbal and non-verbal cues used in face-to-face human conversation (Giles et al., 1991; van Baaren et al., 2004; Stel and Vonk, 2010), as well as verbal and non-verbal cues used in music-making (Dunbar et al., 2012b; Weinstein et al., 2016), may serve to regulate interactions, and facilitate social bonding.

Both music and language have also been traced to antiquity. Archeologists have determined that the size of the vertebral nerve canal – the vertebral nerve itself critical for fine motor control over the musculature related to breathing, as well as the intensity, pitch, duration, and bandwidth of vocalizations – only became equivalent in size to modern humans with the appearance of *Homo heidelbergensis*, about 5 to 800,000 years ago (MacLarnon and Hewitt, 1999; Martínez et al., 2013). The biomechanics of human song production are more demanding than human speech, in several different dimensions, including tidal volume, subglottal pressure range, and muscular control (Sundberg, 1987). If song was in fact a precursor to spoken language, all the necessary neurological and anatomical components may have already been in place and available to serve the function of speech. Perhaps more likely, however, is that the two systems coevolved together (Morley, 2013).

## On the Functional Origins of Music

The origins of the field, often referred to as evolutionary musicology, can be attributed as early as Charles Darwin, first writing in the *Descent of Man* (Darwin, 1871):

*We shall see that primeval man, or rather some early progenitor of man, probably first used his voice in producing true musical cadences, that is in singing, as do some of the gibbon-apes at the present day; and we may conclude from a widely spread analogy, that this power would have been especially exerted during the courtship of the sexes – would have expressed various emotions, such as love, jealousy, triumph – and would have served as a challenge to rivals. It is, therefore, probable that the imitation of musical cries by articulate sounds may have given rise to words expressive of various complex emotions*.

In recent years, this hypothesis of a “musical protolanguage” or “musilanguage” has been offered and repeatedly rediscovered (Brown, 2000; Geissmann, 2000; Marler, 2000; Wallin et al., 2000). Although it is currently unknown how music and language began, these terms suggest a kind of rudimentary form, as well as blurring of the distinction, between modern human music and language. A few plausible evolutionary models have been suggested. Several early and modern theoreticians have speculated that affective, non-referential primate displays emerged at the earliest stage of evolution (e.g., Watts, 2016), a crude “musilanguage” came into play at a later stage (perhaps roughly approximated by modern gibbon-song) (Brown, 2000), which was eventually followed by human song, then instrumental music, and eventually language (Darwin, 1871; Marler, 2000; Dunbar, 2012). A more balanced view suggests that a continuum exists between both music and language, that has likely always existed, dependent on the species and the ecological context. In any case, in terms of an empirical analysis based in comparative animal behavior, “musilanguage” does appear to be present in various extant species, further supporting the claim of a common evolutionary origin and history for both music and language.

Although it is certainly accurate that many species, especially birds, often signal or vocalize in a non-social context, often just the male (Slater, 2000), the discussion here takes social interaction as one of the core defining features of music and language. Human speech does not normally occur in a social vacuum, as conversation is its primary locus of use; similarly, music is typically made in groups, often with other music-making individuals, and traditionally in the presence of an audience. Therefore, comparative analyses which often invoke examples of solitary singing in male birds, primates or other animals, arguably have limited relevance, especially when talking, singing or playing music to oneself are generally viewed as aberrant, preparatory or recreational activities in humans. Group singing is a very common cross-cultural practice, where clusters of people often chant or sing in synchrony (Merker, 2000). Although the focus of this article is on social interaction in the broadest sense, including social groups of all sizes, the most widely practiced form of interactive group vocal behavior in other species, duetting, is generally defined as coordinated singing with overlapped phrasing, typically between two partners of the opposite sex (Haimoff, 1986; Hall, 2004). Though rare cases in which large group “duetting” (i.e., non-dyadic synchronized singing) may exist, which cannot be discounted, the primary focus here is on the well-studied examples of dyadic duetting, that arguably bare the closest resemblance to the synchrony inherent to human music.

In birds, for instance, duetting is typically found between the sexes in a pair of mates in what is generally considered a socially monogamous species (Thorpe and North, 1965; Thorpe, 1972; Wickler, 1980; Farabaugh, 1982). In fact, around 90% of bird families are socially monogamous and of these same families, over 400 species or 40% of bird families practice avian duetting (Hall, 2009). Though correlation does not necessarily imply causation, there are additional reasons to suspect an important relationship between duetting and social bonding. More conclusive findings for the social bonding hypothesis have been discovered comparing species that are closely related, occupying unique ecological niches, in what might be called a “natural experiment.” For example, compared to the sexually dimorphic polygynous red-winged blackbird, duetting in synchrony is done primarily by the monogamous red-shouldered blackbird, appearing to promote a lifelong pair bond (Whittingham et al., 1997).

In non-human primates, duetting has only been observed in a few diverse species, including Old World primates such as spectral tarsiers, Mentawai langurs, indri, and gibbons. Curiously, duetting has only been found in around 10% of primate species, yet in 100% of these known cases, the unusual phenomenon of stable social monogamy has also been observed among mated pairs of these same genera (Haimoff, 1986). Gibbons, for instance, differ from those primates most closely related to humans, the great apes – orangutans, chimpanzees, gorillas, and bonobos – as gibbons are essentially bound to monogamy, due to the extended period of 8–9 years necessary for raising and protecting dependent offspring (Reichard and Boesch, 2003). Further, unlike their polygamous ape cousins, both sexes sing long and complex song bouts, practiced over time to produce significant changes to their vocal repertoires (Haimoff, 1981; Geissmann, 1986; Koda et al., 2013), to create highly synchronized duets for facilitating long-term pair bonds (Geissmann, 1999; Geissmann and Orgeldinger, 2000) (though, it is worth pointing out that solo song often seems to function as pair-bond advertisement even in non-duetting gibbons) (Ham et al., 2017). Pairs of gibbons that engage in the most synchronized songs generally allocate more time to the same activities, reciprocal grooming, and occupying the same spatial proximity (Geissmann and Orgeldinger, 2000). The synchronized vocal behavior of spectral tarsiers, Madagascan indris, and Mentawai langurs exhibits a similar pattern (Haimoff, 1986; Gamba et al., 2016). Finally, recent studies have further discovered that even among the non-monogamous chimpanzee, the occurrence of pant-hoot chorusing reflects both short-term, and long-term social bonding within the highly fluid fission-fusion social dynamics of chimpanzee societies (Fedurek et al., 2013). Like those interactions that occur in both human conversation and music-making, gibbons, indris, and chimpanzees appear to be capable of adjusting the timing, unit duration, interval duration (Fedurek et al., 2013; Koda et al., 2013) and even pitch variation (Torti et al., 2013; Gamba et al., 2016) of such emissions. The occurrence of duetting, chorusing and other similar acoustic features in these species strongly suggests an instance of convergent evolution, as an adaptation to a similar ecology and social structure for the function of social bonding (Haimoff, 1986; Fedurek et al., 2013; Gamba et al., 2016). Perhaps unsurprisingly, duetting in various other mammals, including elephants, dolphins, whales, bats, and naked mole-rats further seems to be related to social bonding (Filippi, 2016).

In addition, music-making in other species does not appear to be solely restricted to the vocal modality. In particular, periodic beating on sonorous objects – also known as drumming – has recently been discovered in chimpanzees (Goodall, 1986; Arcadi et al., 2004; Nishida, 2011), gorillas (Schaller, 1963; Redshaw and Locke, 1976), and bonobos (de Waal, 1988; Kugler and Savage Rumbaugh, 2002), implying that this potential predecessor of instrumental music evolved perhaps 10 million years ago, in our last common ancestor (Fitch, 2015; Kotz et al., 2018). In chimpanzees, drumming patterns seem to act as individually distinctive long-distance signals that function to coordinate the grouping dynamics among fission-fusion chimpanzee societies (Babiszewska et al., 2015): in effect, helping to facilitate social bonding. In summary, emerging evidence suggests that the impulse to coordinate and synchronize behavior, including vocal behavior, may be an important facilitator of bonded relationships in a variety of different species, including humans (Dunbar and Shultz, 2010).

## Music and Social Bonding in Humans

In non-human primates, social connections are also created, and maintained through dyadic social grooming, which activates endogenous reward-producing endorphins, further promoting social bonding (Keverne et al., 1989; Machin and Dunbar, 2011). In humans, endorphins have also been implicated in the motivational reward system for several synchronized social activities, which are also socially bonding, such as laughter (Dunbar et al., 2012a), rowing (Cohen et al., 2010), and exercise (Davis et al., 2015).

Music-making is also often synchronized or tightly coordinated, in activities such as group drumming (Dunbar et al., 2012b), communal singing (Weinstein et al., 2016), and dance (Tarr et al., 2016) which seems to have an important influence on social bonding. Other recent studies have shown that joint music-making, that involves group singing, drumming, and dancing, also seems to promote cooperation in small children (Kirschner and Tomasello, 2010). Moreover, these effects do not seem to be limited to active, interpersonal musical participation, as simply hearing music from a particular culture can elicit affiliation toward people from that same culture (Vuoskoski et al., 2017), showing that music can also be an important part of personal identity formation (Hargreaves et al., 2002). In fact, just the mere act of listening to music activates the pleasure-inducing dopamine reward system (Ferreri et al., 2018), as well as the motor cortex in the brain (Zatorre et al., 2007; Grahn, 2012), revealing an important biological connection between music, reward and movement, perhaps further suggesting an evolutionary link between music and dance. Indeed, traditional societies both historic and modern, typically perform music and dance in an integrated social context (Mackrell, 2019). However, it is currently unknown whether synchronized or coordinated guitar, flute, piano or other forms of instrumental music-making also promote social bonding, possibly due to the reward of these activities. An important goal of emerging research is to determine if alternative musical activities also promote social bonding, and if so, which factors are most critical for helping to facilitate this process, such as the presence of an external beat, rhythm, physical exertion, synchrony, and endogenous opioids (Tarr et al., 2014).

## On the Functional Origins of Language

The synchrony which is often characteristic of music, and its general scarcity in speech, illustrates one of the clearest differences between music and language. The duet singing found among many non-human animals appears to be at least partially if not fully synchronous. In most species of gibbons, for instance, the pair alternates their calls while duetting, albeit with significant overlapped phrasing (Geissmann, 2002). However, a rare class of truly antiphonal vocalizations, which seems to bridge the gap between animal calls and human speech, has been referred to as cooperative vocal turn-taking (Levinson and Holler, 2014; Levinson, 2016; Pika et al., 2018). To date, cooperative vocal turn-taking has been found in a small number of diverse social animals, including primates, dolphins and elephants, often expressed in cases of approach, and integration into a social group or existing strong social relationships (for a review, see Pika et al., 2018). In primates, this back-and-forth mode of communication has been most clearly documented in cooperative breeding marmoset monkeys and humans (Ghazanfar and Takahashi, 2014). Moreover, like cooperative vocal antiphony in pair-bonded or group-bonded species, cooperative vocal antiphony in polygynous primate societies also seems to happen primarily between affiliated conspecifics (Symmes and Biben, 1988).

Curiously, humans and marmosets are included in a select number of primates that create long-term pair bonds and display cooperative breeding from both parents (male parental investment is minimal or absent in most primate species) (Zahed et al., 2008). In marmosets and humans, the prosocial impulses and cognitive processes which motivate cooperative breeding have been extended to other unrelated individuals, encompassing social attention, social signaling, social coordination, and social tolerance (Burkart and van Schaik, 2010). Notably, this degree of prosociality beyond closely related kin is not generally observed in other primates, apart from bonobos, which incidentally, exhibit vocal exchanges driven by social bonding (Levréro et al., 2019), highly synchronized male-female duets (de Waal, 1988), and loud calls which function for group coordination (White et al., 2015), all of which are conspicuously absent in their closely related, but decidedly less prosocial chimpanzee cousins (Haimoff, 1986; Ujhelyi, 2000). Importantly, marmosets will participate in long-distance cooperative vocal turn-taking with unseen individuals (Miller and Wang, 2006; Chen et al., 2009), according to a temporal structure that parallels the turn-taking process that humans use in typical conversations (Stivers et al., 2009). Finally, cooperative vocal turn-taking in marmosets appears to be guided during ontogeny through parental reinforcement, indicating the presence of vocal learning (Chow et al., 2015). Although original estimates suggested a lack of vocal learning in non-human primates (Owren et al., 1992; Janik and Slater, 1997), more recent analyses appear to indicate the presence of often limited, and sometimes complete vocal learning in many non-human primate species (Haraway and Maples, 1998; Hopkins et al., 2007; Snowdon, 2009; Petkov and Jarvis, 2012; Koda et al., 2013; Chow et al., 2015). Due to these striking parallels between marmosets and humans, this form of communication has been suggested as an instance of convergent evolution, as coordinated vocal turn-taking is not typically seen in other primates (Ghazanfar and Takahashi, 2014).

More broadly, the social complexity hypothesis demonstrates that complex social species require sophisticated communication in order to manage encounters within the group and promote social cohesion (Freeberg et al., 2012). Notably, vocal repertoire size seems to be at least somewhat contingent on the capacity for vocal learning, mainly evolving in a small fraction of organisms (often limited to socially monogamous species, where social bonding is important) including songbirds, gibbons, and humans (Geissmann and Orgeldinger, 2000; Koda et al., 2007; Dallmann and Geissmann, 2009; Snowdon, 2009; Petkov and Jarvis, 2012). Humans appear to be no exception, not differing *qualitatively* but only *quantitatively* in this respect. This is an unsurprising fact, given the human proclivity for serial social monogamy necessary for raising dependent offspring (Burleson, 2015), friendships with unrelated others (Oesch, 2018), and unprecedented vocabulary-size of over 60,000 words for the average adult speaker (Pinker, 1994), quirks all unique to humans. This further suggests that the flexibility inherent to vocal learning may be central for prosocial or socially bonded species. In contrast, for species in which innate, fixed calls predominate, simpler vocalizations may be adequate for less prosocial functions, such as territorial defense, and alarm-call signaling (Seyfarth et al., 1980; Blumstein, 1999).

In fact, this clear dichotomy between flexible, volitional vocal learning versus innate, fixed calls has led several theorists to argue that two different motor systems are needed to produce human speech (Hage and Nieder, 2016; Holstege and Subramanian, 2016). For example, Hage and Nieder (2016) have recently suggested a dual neural network model: (1) a volitional articulatory motor network (VAMN), based in the prefrontal cortex and Broca’s area, controlling words and sentences, and (2) a primary vocal motor network (PVMN), situated in older, phylogenetically conserved subcortical structures, controlling laughter, cries, and the expression of emotions. Indeed, recent MRI studies reveal an expansion of cortical auditory-vocal (Ardesch et al., 2019) and sensori-motor connectivity in the human brain over other primates (Kumar et al., 2016), which were likely instrumental to the enhancement of vocal working memory and vocal repertoire size in humans (Aboitiz, 2018).

## Language and Social Bonding in Humans

Synchronized speech has long been a common form of ritualized behavior, both in traditional societies, as well as modern industrial societies (e.g., simultaneous praying, chanting, and pledging) (Stolba, 1994), and appears to facilitate social bonding (Zimmermann and Richardson, 2016). In spite of the fact that coordinated antiphonal turn-taking is the norm for typical conversations, linguistic aspects of synchrony are apparent even within typical turn-taking conversations (Cummins, 2003). More specifically, when people converse, they often merge linguistically on many features of the conversation, from lexical items, phonology, speech rate, pitch, pause rate, variants specific to different regions, pause duration, body language, and grammar (Pardo, 2006; Adank et al., 2013; Lev-Ari, 2016; Levinson, 2016). One major factor argued to influence this convergence is a desire to establish rapport or increase affiliation with a conversation partner (Giles et al., 1991; van Baaren et al., 2004; Stel and Vonk, 2010; Pardo et al., 2012; Manson et al., 2013) and to increase conversation success (Pickering and Garrod, 2004). For example, several linguistic and behavioral similarities often expressed during conversational interactions, including selective grammatical convergence (Lev-Ari, 2016), and accent imitation (Adank et al., 2013), facilitate affiliation between speakers. Similar findings have been reported in the context of romantic relationships. For instance, both romantic commencement, and later relationship stability are positively predicted by language style matching (LSM) scores in conversation (calculated as a composite measure of similar verbs, nouns, pronouns, and articles used in interaction) (Ireland et al., 2011).

Conversational content is also a significant facilitator for the social bonding effects of language. Analyses of actual human conversational content, both in industrial Western cultures as well as more traditional societies, reveal that social gossip topics overwhelmingly consume most conversation time (Haviland, 1977; Dunbar et al., 1997). Notably, the ubiquity and frequency with which humans engage in social gossip has been shown to have non-trivial functional significance within the broader social network of relationships, beyond mere idle chatter. Weaver and Bosson (2011) found feelings of interpersonal closeness were facilitated through social gossip; in particular, the effects seemed to be strongest when participants shared a decidedly negative perspective on an absent person. Neurophysiological studies have further noted an increase in salivary oxytocin levels during such conversations, suggesting a hormonal correlate of gossip behavior that facilitates social bonding (Brondino et al., 2017). In addition, the psychopharmacological component of social bonding has been linked to additional aspects of conversation beyond mere gossip; specifically, neuroimaging studies have shown increased activation in the mesolimbic dopamine reward system of the brain linked with self-disclosure to close family and friends (Tamir and Mitchell, 2012). Thus, the inclination to convey inner thoughts to others may confer an adaptive benefit by promoting social bonding. This is particularly noteworthy, as only a handful of activities, apart from food, sex, and others with clear survival value, have generally been found to stimulate the reward system in the brain (Berridge and Kringelbach, 2015).

Moreover, preliminary findings seem to suggest that the mere sound of voices that are familiar may have a similar affect, comparable to the way in which familiar, trustworthy faces also activate the reward system (Sánchez-Adam et al., 2013). Abrams et al. (2013) has shown that autistic children have reduced activity between brain regions linked with speech perception and reward processing, reflecting their relative lack of engagement with speech in daily life. More directly, other studies have shown increased oxytocin activity (Seltzer et al., 2010), and lowered cortisol levels when children hear their mother’s voice (Seltzer et al., 2012). Given that familiar faces are an important cue of the in-group and have also been shown to activate the reward system (Sánchez-Adam et al., 2013), reward associated with familiar voices, as a similar cue of the in-group, may also motivate social bonding (McGettigan, 2015).

## Pitch, Tone, Prosody and Emotional Expression in Human Speech, and Music

Tone is the use of pitch in language to distinguish lexical meaning with respect to particular words. Languages that make considerable use of tone, as a suprasegmental linguistic feature, are known as tonal languages. Though often viewed as oddities by linguists, tonal languages, in fact, make up 60–70% of the world’s languages (Yip, 2002). Given this rather curious fact about language, some theorists have argued that modern language itself first originated as a tonal language, and that the evolution of intonation languages later happened as a result of the loss of lexical tone (Brown, 2000). Evidence in support of this view comes from the fact that most African languages – Africa being the ancestral location where the origin of our species, and presumably language, arose (Atkinson, 2011) – are in fact tonal languages (Maddieson, 2013). Modern examples of intermediate cases also exist, known as pitch-accent languages, such as Swedish, Japanese and Serbo-Croatian, where some degree of tone is used amid intonation (Brown, 2000). Nonetheless, the central point is that tonal languages represent the normative, and probably the ancestral case of language (Snowdon et al., 2015).

Other examples attest to the close connection between music and language. For instance, the structure of human speech sounds, including vowel sounds, predicts the architecture of the chromatic scale as well as the ordering of consonant pitch relationships (Schwartz et al., 2003; Snowdon et al., 2015). Another recent study analyzed speech and music from three tonal and non-tonal languages and discovered that larger and more frequent changes in pitch direction occurred in both the music and language of cultures with tonal languages, perhaps suggesting a coevolution of music, and language (Han et al., 2011). Thus, harmonic speech structures nearly mirror that of harmonic music structures cross-culturally, and visceral modulations in emotion are apparent in different harmonic speech structures, similarly to music (Gill and Purves, 2009; Bowling et al., 2010).

Prosody, or the stress and intonation patterns of an utterance, is another music-like aspect of language. In conversation, prosodic aspects like pitch or word elongation at the end of an utterance are often used for the coordination of turn-taking and rhythm determination in conversation between interlocutors (Ward and Tsukahara, 2000; Ten Bosch et al., 2005; Levinson, 2016). In fact, at least one recent study indicates a strong universal standard for turn-taking behavior in conversation (within 200 ms of the end of each utterance), cued by prosodic features across many of the world’s languages, suggesting a biological basis for timings in response to speakers (Stivers et al., 2009). Humans also seem to distinguish affective vocalizations more obviously through intonation and pitch than through the words themselves. Between parents and prelinguistic infants, specific prosodic features of language have been shown to influence infant behavior (Fernald, 1992). For instance, shorter, rising upward staccatos typically create arousal, while longer, descending intonations generally have a calming influence. Developmental studies also suggest the song-like quality inherent to child-directed speech is likely related to social bonding and the solicitation of parental investment (Feldman et al., 2011).

Curiously, the expression of emotion through voice does not seem to be unique to humans, and the auditory features involved appear to have a similar influence on the anatomy and physiology of animal receivers. For instance, a similar phenomenon seems to occur in the whistles and calls used by people to influence domestic animal behavior, such as the actions of horses and dogs, suggesting that these same signals are effective across species (McConnell, 1990, 1991). Although it is currently unknown how and why non-human animals respond in this way to human vocalizations, the most parsimonious explanation might suggest that they are conditioned to respond in certain ways to specific signals, as opposed to intuiting a more profound cross-species meaning to these calls. Nonetheless, it remains an open question, and given the preceding discussion, the social bonding hypothesis should perhaps not be discounted. Finally, several studies have found notable similarities between speech and music in the accuracy with which listeners can identify discrete emotions in both domains (Juslin and Laukka, 2003).

In summary, speech and music show a close interconnection with respect to prosody, tone and pitch, and the connection is most obvious with respect to emotional communication. Both the prosody of music and speech and the continuous sequence of music and speech sounds convey affective semantics. These same features are influential in changing human infant behavior, as well as the behavior of domestic animals, suggesting that these same affective vocalizations are influential across species (Snowdon et al., 2015). Given that prosodic changes in vocalizations can communicate affective information and organize vocal interactions among many individuals across a wide range of species, the capacity for affective, and interactional prosody may have facilitated the evolution of musical and linguistic prosody (Filippi, 2016).

## Discussion

Steadily emerging evidence suggests a unique association of traits unique to both human music and language. Perhaps most critically, studies of human social behavior suggest an important function for synchronized music and antiphonal speech, while studies of animal social behavior suggest an important function for synchronized and antiphonal vocalizations, both in the context of social bonding.

A potential objection to the arguments and evidence presented here is that social bonding is unlikely to be the primary adaptive function for the presence of song and other complex vocalizations in birds, non-human primates, and other mammals or for the existence of music and language in modern humans. In other words, the motivation to bond might have been supportive of the evolutionary process but is unlikely to be the sole explanation. An alternative explanation is that signaling, or the exchange of information may have been the main driver for the evolution of music (Hagen and Bryant, 2003; Reddish et al., 2013) or language (Pinker and Bloom, 1990). However, this objection ignores several key constraints that have been argued to be critical for any hypotheses that address the evolution of music and language (Merker et al., 2015).

The first constraint for any proposal which claims to account for the evolution of music must explain the cross-cultural inclination for occasional group singing and dancing (Brown, 1991; Brown and Jordania, 2013). Communal singing is especially prevalent among humans, with groups of people often singing or chanting in synchrony with one another (Merker, 2000). Moreover, communal singing in large groups has further been shown to indeed bond large groups of people (Weinstein et al., 2016), which is consistent with this first constraint as well as the social bonding hypothesis, but inconsistent and not predicted by the information exchange hypothesis (which, incidentally, might predict a sort of dyadic turn-taking style of information-rich “song exchange” in humans).

The second constraint for any proposal which attempts to explain the evolution of music or language must have within it the ability to explain the human evolutionary trend toward reduced sexual dimorphism, as well as increased pair-bonding, altriciality, and social group size (Klein, 2009). Not to mention, the development of these traits has had a direct influence on the evolution of further behaviors related to signaling, display, sexual selection, cooperative breeding, vocalizations between adults (Merker, 2012), and vocalizations between infants and adults (Falk, 2004). The only known proposal which is consistent with this second constraint is the social brain hypothesis (Oesch, 2018), and by extension, the social complexity hypothesis for animal communication (Freeberg et al., 2012; Oesch, 2018), which predicts and finds a relationship between complex vocalizations and social bonding. This second constraint further demands that any alternative proposal which argues for a modification of ancestral “carnival displays” (i.e., a group noise-and-movement display in chimpanzees) in early hominins (Merker et al., 2009), coalitional displays in humans (Hagen and Bryant, 2003), or size-exaggeration vocalizations, must be embedded within this ecological context (Morley, 2013). Furthermore, a display or signaling hypothesis must first be explicit about what exactly is being displayed, and then must explain how this thing came to be in the first place. For example, coalitional signaling is in fact signaling the presence of a bonded coalition, which presumably became bonded in the first instance via the influence of language, music, song, and other cultural activities. In other words, if the coalitional display hypothesis is unable to rule out music, language, song or other complex vocalizations as the origin for a coalition in the first place, then the hypothesis is a non-starter as a primary driver for the evolution of these traits.

The third constraint for any proposal which attempts to explain the evolution of music must have within it the ability to explain synchronization or entrainment. In this case, the social bonding hypothesis once again suggests a plausible explanation. One possibility is that a beat may aid participation and coordination of a performance, especially among large groups, whereas an unregulated song might tend to dissolve into a noisy cacophony of uncoordinated voices (Geissmann, 2000). In contrast, among dyads, one has only to synchronize to one other individual, rendering a beat unnecessary. This hypothesis also fits well with three facts we already know about the social context of song production in non-human animals, human evolution, and the social context of song production in humans: (1) in the case of group duetting, birds, gibbons and other species typically constrain their duets within the context of dyadic pair-bonded relationships (Geissmann, 1999; Geissmann and Orgeldinger, 2000; Hall, 2009), (2) social groups increased in size over the course of human evolution (Oesch, 2018), and (3) in humans, song is often made communally in large groups of people (Merker, 2000). Once a close-knit group has been formed, through the social bonding effects of language, music or song, the introduction of a steady beat, such as through synchronized drumming (Dunbar et al., 2012b) or marching (Wiltermuth and Heath, 2009), allows a large group to synchronize their song or music to an external time-keeper, enhancing the perception of internal group cohesion (Fessler and Holbrook, 2014). Of course, once the performance of song or rhythm-based music is underway, this may further serve as a coalitional display, or honest signal (Zahavi and Zahavi, 1997; Pentland, 2010), of social cohesion to external predators or groups which could present a threat, especially in times of warfare (Hagen and Bryant, 2003; Hagen and Hammerstein, 2009). As far as the individuals that make up socially bonded groups face improved odds of survival over groups of individuals which are less bonded, behaviors which promote social bonding, reciprocity, and trust can be considered adaptive (Dunbar and Shultz, 2010).

Despite the identification of these constraints, and a preponderance of evidence in support of the social bonding hypothesis, some theorists have nevertheless argued, that were it not for the adaptive significance of signaling or the exchange of information, neither speech, song, or music would have evolved the capacity to convey rich and complex data (Pinker and Bloom, 1990). However, this is a non-sequitur for at least two reasons. First, the only proposal to date that accounts for the evolution of repertoire size in humans, non-human primates, and various other species, is the social complexity hypothesis for animal communication, which demonstrates a relationship between vocal repertoire size and social bonding (Freeberg et al., 2012). Second, following directly from this first point, the capacity to bond through vocalizations is not devoid of “information.” As all animal communication systems convey “information” (Bradbury and Vehrencamp, 2011), of one sort or another, the central issue is what *kind* of information and for what *function* an information-rich communication system evolved. In the case of human language, the capacity to bond via gossip, and through self-disclosure to family and close friends depends profoundly on the ability to transmit complex information. Indeed, there is no more reason to expect that social bonding requires the transmission any less linguistic “information” than cultural learning (Pinker and Bloom, 1990).

Nonetheless, despite a preponderance of evidence in support of the social bonding hypothesis, it would nonetheless be a fallacy to conclude that social bonding is the *only* function of song, music, complex vocalizations, or language in humans or other species. In fact, most evolutionary biologists make a distinction between the *ancestral* and *derived* traits (Ridley, 2004), or as they are sometimes referred to in evolutionary linguistics and musicology, as the *direct* and *derived* traits or functions (Millikan, 1984; Origgi and Sperber, 2000). Specifically, a direct function refers to the ancestral primary driver for which a trait arose, while the derived function(s) refers to the way in which the trait may later have been modified or co-opted for additional secondary purposes. For example, in the case of animal communication, Hall (2004) reviewed an assortment of hypothesized functions for duetting, including relationship commitment, signaling mated status to rivals, and ownership of the physical territory and/or advertisement. All three proposals had some evidence-based support. Yet, one could still make the case that social bonding is in fact the ancestral or direct function: relationship commitment, signaling mated status to rivals, and ownership of the physical territory could all be interpreted as various indications of the motivation to defend this same previously existing pair-bond. In the case of human language, several functions have also been proposed, including social bonding (Oesch, 2018), sexual advertisement (Oesch, 2017), and deception (Oesch, 2016), all of which have some degree of evidence-based support. Therefore, it would also be incorrect to conclude that each of these hypotheses are mutually exclusive. Here, it is argued that social bonding was the original ancestral function for which both human language and music evolved, while sexual advertisement, cultural learning, signaling, and others may be derived functions, interacting and expressed in different ways, depending on the ecological context (Merker et al., 2015; Pika et al., 2018).

To elaborate, it is important to recall that both music and language consist of many different aspects in both processing and production. In the case of music, music is composed of rhythm, musical pitch, melody, and several other aspects. In the case of language, language is composed of syntax, semantics, pragmatics, phonology, phonetics, prosody, and many other features. Bearing this in mind, some theorists have argued that many of these features may have originally evolved independently of music or language, only later to be co-opted by music, language, or both domains for different reasons (Fitch, 2006). Although it is difficult to speculate at which point during human evolution certain aspects of music or language may have been co-opted for different functions, depending on different social contexts, there is certainly good evidence that not all aspects of music or language function only for social bonding (Leongómez et al., 2014, 2017; Pisanski et al., 2018). For instance, in the case of speech prosody, both men and women have been found to modulate their voice pitch in courtship situations in order to elicit favorable attention from desirable prospective mates (Leongómez et al., 2014; Pisanski et al., 2018). Other studies have shown that individuals modulate the parameters of their voice, specifically fundamental frequency (F_0_), according to their own self-perceived social status, as well as the perceived social status of the listener (Leongómez et al., 2017). Based on such findings, some authors have argued for a common origin of music and language based primarily in sexual selection (Leongómez et al., 2014). Yet, apart from these few paralinguistic features of language, the sexual selection account has thus far not been able to explain what are generally considered to be the most critically defining linguistic features of language (e.g., syntax, semantics, pragmatics, and phonology) and music (e.g., rhythm, musical pitch, and melody).

A dissimilar, but related objection, claims that the first forms of communication were gestural, rather than vocal, while the first forms of speech were a late emergence in human evolution. However, the commonalities in vocal behavior between human and non-human primates, as well as the many close links between speech and song, suggest a long, gradual evolution of vocal behavior in our ancestors. Thus, although the earliest forms of communication may have been both vocal and gestural (e.g., song and dance), it seems likely that speech eventually became the predominant mode of communication, while gestures supported speech, just as they do now in modern humans. In summary, vocal communication has a long evolutionary history that proponents of the gestural theory of language origins tend to ignore or devalue, while not considering the comparative and human evidence to the contrary.

A final objection worth addressing is that the partial attention to dyadic interactions in human and non-human animals is too narrow a focus for an article which claims social interaction of all group sizes, as central to the study of music, and language. While it is true that the focus here, for many of the examples which come from birds, humans and non-human primates, has been on dyadic interactions, this was due to limitations of the current literature, not active choice. Although unresearched cases in nature may exist, a quick survey of the literature reveals few documented cases of group synchronous or antiphonal behavior beyond dyadic interactions. It is hoped that this review will simulate further empirical research on larger social groups, beyond dyadic interactions, for evidence of large group duetting, musical, or turn-taking behavior. If such cases were to be found, one prediction worth investigating is whether the coordination presumably inherent to such interactions was kept synchronized according to some beat, rhythm, or external timepiece (i.e., in this sense, directly analogous to human music). In fact, the so-far reported paucity of examples of non-dyadic duetting, instrumental music, or antiphonal vocalizations in nature, could be interpreted as indirect support for the social bonding hypothesis. In other words, it is probably human groupings alone that have become so large, over the course of human evolution, and the necessity for these groupings to operate in a cooperative, socially bonded fashion, as to require social bonding agents like music, dance, language, and laughter.

## Conclusion

Music and language are universal human capacities with many commonalities, especially with respect to their acoustics and structure, social interaction, social evolution, and functional origins. Studies of animal social behavior suggest an important function for synchronized and antiphonal vocalizations, both musical, and non-musical in the context of social bonding. Similarly, studies of human social behavior also suggest an important function for synchronized music and antiphonal speech in the context of social bonding. In contrast to an often-touted view, which treats music as an immaterial object of frivolous acoustic pleasure (Pinker, 1997) or language as a faculty for information transmission (Pinker and Bloom, 1990), this proposal might instead explain how music and speech connect us to other people. If music and speech are in fact fundamentally social, this could have profound socio-economic implications for how to consider the function of music and speech in our modern world, based on our evolutionary history.

Indeed, the contemporary urban social environment holds an inherent contradiction. We live in large unfamiliar communities, but our familiar social networks are very small: around ∼150 according to Dunbar’s number (Oesch, 2018). This tension between small-scale prosociality and large-scale individualism may be central to urban dysfunction. However, if music and language evolved for social bonding, this may inform business and organizational structure, digital communication design, and online and offline social networks. Moreover, the multi-disciplinary approach necessary for understanding this overlap between music and language may facilitate a more integrated social sciences that can equal the mainstream sciences.

That said, the frequent difference between the synchrony of music and antiphony in spoken language raises critical questions for hypotheses proposing a similar origin for these domains based in social bonding (Brown, 2000; Marler, 2000; Merker, 2000; Merker et al., 2009). Namely, did the presumed ancestral musilanguage consist of continuous, synchronous beats? If so, why has this aspect been lost in modern speech? If not, why has this feature emerged in music? Stated more technically, what fitness advantage might there have been, during human evolution, to add a steady beat to a song vocalization or to music in general? In other words, if non-rhythmic animal song is enough for bonding non-human animals, why does human song and song-infused music generally have a rhythmic, steady beat? Although this first issue is hard to answer definitively, hints to the latter four queries, as has been noted, may come from taking into account the social context where continuous temporal patterns often happen. Namely, once a group has been formed, via the bonding influence of language, music or song, a very plausible hypothesis is that the introduction of rhythm allows a group to synchronize this same song or music to an external time-piece, enhancing the perception of internal group cohesion (Fessler and Holbrook, 2014), and conveying an honest signal (Zahavi and Zahavi, 1997; Pentland, 2010) of external group cohesion to predators or rival human groups (Hagen and Bryant, 2003; Hagen and Hammerstein, 2009).

An important project for future research is to use the synchronous and antiphonal paradigm to describe and compare the behavioral phenotypes from a wide spectrum of primates. In principle, this could be achieved by a methodical and selective investigation the over 200 distinct primate species. In so doing, this should enable us to create cladograms that describe the evolutionary history of primates’ synchronized and turn-taking communication systems (Pika et al., 2018). Additionally, few studies have examined the influence of synchronization or turn-taking on social bonding for alternative forms of music-making in humans, apart from drumming or singing, or examined the influence of synchronization or turn-taking on social bonding for other social activities, such as conversation, despite earnest calls to do so (Tarr et al., 2014). Moving forward, this is an important avenue for future research.

## Data Availability

No datasets were generated or analyzed for this study.

## Author Contributions

NO wrote, edited, and approved the final version of the manuscript.

## Conflict of Interest Statement

The author declares that the research was conducted in the absence of any commercial or financial relationships that could be construed as a potential conflict of interest.
